# Artificial Intelligence in Dentistry—Narrative Review

**DOI:** 10.3390/ijerph19063449

**Published:** 2022-03-15

**Authors:** Agata Ossowska, Aida Kusiak, Dariusz Świetlik

**Affiliations:** 1 Department of Periodontology and Oral Mucosa Diseases, Medical University of Gdańsk, 80-204 Gdańsk, Poland; agata.ossa@wp.pl; 2 Department of Biostatistics and Neural Networks, Medical University of Gdańsk, 80-211 Gdańsk, Poland; akusiak@gumed.edu.pl

**Keywords:** artificial intelligence, neural networks, dentistry

## Abstract

Nowadays, artificial intelligence (AI) is becoming more important in medicine and in dentistry. It can be helpful in many fields where the human may be assisted and helped by new technologies. Neural networks are a part of artificial intelligence, and are similar to the human brain in their work and can solve given problems and make fast decisions. This review shows that artificial intelligence and the use of neural networks has developed very rapidly in recent years, and it may be an ordinary tool in modern dentistry in the near future. The advantages of this process are better efficiency, accuracy, and time saving during the diagnosis and treatment planning. More research and improvements are needed in the use of neural networks in dentistry to put them into daily practice and to facilitate the work of the dentist.

## 1. Introduction

Nowadays, artificial intelligence (AI) is becoming more important in medicine and in dentistry. It can be helpful in many fields where the human may be assisted and helped by new technologies. The developments in AI started in 1943 but the term “artificial intelligence” was created in 1956 at a conference in Dartmouth by John McCarthy. Machine learning, neural networks, and deep learning are subsets of artificial intelligence. Machines can learn through data to build algorithms and in this way, they can solve the prediction problems without human help. Neural networks (NNs) use artificial neurons that are similar to human neural networks and mimic the human brain in a mathematical non-linear model. NNs are able to simulate human cognitive skills such as problem solving and human thinking abilities, which includes learning and decision making. Neural networks in a simple form have three layers: input layer (where the information enters the system), the hidden layer (where the data are processed), and the output layer (where the system decides what to do). Given a set of mathematical models, NNs are able to outline any input to an output. If an adequately large amount of data are available, such NNs can be trained to represent the intrinsic statistical figures of the provided data. The topology of the simple artificial neural network is shown in Figure 3 in Swietlik D et al. (2004). There are also more complex artificial neural networks where there are more hidden layers and these are called multilayer perception (MLP) neural networks. The most commonly used types of neural networks are artificial neural networks (ANN), convolutional neural networks (CNN), and recurrent neural networks. Deep learning is a part of neural networks where the computer learns on its own how to process the data. Deep learning neural networks have between a few thousand and a few million neurons in the hidden layer [1,2,3,4,5]. Artificial intelligence (AI) may be used in planning more effective therapies, prophylaxis, and the reduction in treatment costs [2,4]. We can benefit from AI in medicine, mostly in the fields such as radiology, pathomorphology, and oncology (by using “Thermalitics” technique in breast cancer detection), in cardiology (to help in ECG analysis), in psychiatry (to diagnose, prevent and treat mental illnesses), nuclear medicine, and many others [6,7,8,9,10]. The computer models of neural networks are also one of the methods to understand the functioning of the nervous system, which we cannot study in natural conditions due to the limitations of modern research methods [11,12,13,14,15]. 

Artificial intelligence is also spreading in dentistry due to the technological advancements and digitization of dentistry. Dental second opinions can now be made by computers in many dental fields. NNs in dentistry can be used to make the process of diagnosis more accurate, rapid, and efficient. Fast development and new studies related to neural networks in dentistry were the reason to provide this narrative review. The aim of this study was to outline the overall picture of the possibilities of using neural networks in modern dentistry.

## 2. Neural Networks in Restorative Dentistry

Dental caries is the most common dental disease and that is why its disclosure in the early stage is crucial. For the screening and diagnosis of dental caries, dentists mostly use dental probes, and through the observation of the texture and discoloration, they can determine whether the tooth is sound or not. This method is very subjective and is based on the dentist’s experience. In particular, the approximal surfaces may be problematic in dental examination [16,17]. Additional tests such as radiographs are essential in modern dentistry and can enhance the detection of caries. The most common types of radiological images used in caries screening are bitewings, periapical X-rays, and panoramic X-rays. CBCT is used less frequently in tooth decay detection [18,19]. Dental caries detection on radiological images might be assisted by neural networks, which makes the examination faster and more precise. Neural network use in conservative dentistry has developed quickly, but is not very widespread yet [20]. Algorithms can be used to locate the edges of anatomical and pathological structures, which might be very similar to each other due to the image noise and low contrast [21]. In the work by Geetha et al., an artificial neural network was used to determine whether there were caries or not in the 105 radiograph images. They extracted sixteen feature vectors from the segmented image and these were the input nodes. There were two output nodes that consisted of caries or sound tooth. The accuracy of caries detection was 97.1%, and the false positive rate was 2.8%. This study indicates that neural networks may be much more precise in tooth decay detection than traditional dental examination [22]. Moreover, dental restorations may be revealed by the use of artificial intelligence. In restorative dentistry, AI can be used to detect and classify dental restorations such as in Abdalla-Aslan R et al.’s research from 2020. The algorithms used in their work detected 93.6% of dental restorations on 83 panoramic images. Additionally, restorations were classified into 11 categories by using the shape and distribution of grey values [23]. Neural networks might be helpful in planning the selection of the dental treatment and cavity preparation technique. Artificial neural networks were used in Javed et al.’s study to predict the post-Streptococcus mutans prior to dental caries excavation based on pre- Streptococcus mutans using an iOS App developed on an artificial neural network (ANN). For the research, 45 primary molars with occlusal caries were used. The colony forming units for pre- and post-Streptococcus mutans were recorded. The study demonstrates that ANN can predict which excavation method is the best for an individual patient. The accuracy of ANN was 99.03% and it was microbiologically checked (Table 1). The prediction of post-Streptococcus mutans avoids the examination of post-Streptococcus mutans, re-excavation, and re-examination, and pulpal trauma with the excavated cavity [3].

Neural networks in restorative dentistry may be used in a few clinical purposes. Frequently performed diagnosis and the choice of treatment method can now be assisted by artificial intelligence. The most common way to engage new technologies is the analysis of dental X-rays and caries or restoration detection, but also in other fields such as microbiology, which can be assisted by neural networks to make the best treatment decisions. More studies need to be performed to introduce new technologies to daily practice but other fields of restorative dentistry might also be helped by neural networks in decision-making.

## 3. Neural Networks in Endodontics

Artificial intelligence has an increasing relevance in endodontics. It can be useful in detecting periapical lesions and root fractures, root canal system anatomy evaluation, predicting the viability of dental pulp stem cells, determining working length measurements, and predicting the success of retreatment procedures [24]. Artificial neural networks may be used as a decision-making system for locating the minor apical foramen on radiographs. In Saghiri et al.’s research, endodontic files were used to determine the length of the canals on the radiology images with the use of artificial neural networks and without. The measurements were taken before the extraction of the teeth and after the extraction with the use of stereomicroscopy. The correct assessment made by the endodontics was strict in 76% and by the artificial neural network in 96% (Table 2). This shows that artificial neural networks may be used to assess the localization of apical foramen more precisely than humans [25]. Apical periodontitis is an inflammatory process mainly caused by the bacterial infection of the root canal system. It may be detected through radiographic diagnostics and manifest as periapical translucencies that are also named periapical lesions. To reveal periapical translucencies, most are taken as periapical or panoramic radiographs and cone-beam computed tomographic images [26,27]. Setzer et al. in their research used deep learning to detect periapical lesions on cone-beam computed tomographic (CBCT) images. The accuracy of finding the lesions was 93% [25,27]. CNN was also used in Orhan et al.’s work to detect periapical lesions on CBCT images. The convolutional neural network detected 142 of 153 periapical lesions (92.8% accuracy). The results obtained by CNN were similar to those obtained by an experienced dental practitioner [28]. Convolutional neural network (CNN) is a specialized kind of artificial neural network that is very useful when extracting features from the image by engaging convolutional operations. These convolutional neural networks were used in Pauwels et al.’s work. The periapical radiographs were evaluated to find periapical lesions made in bovine ribs. The results were compared with three oral radiologists and the CNN showed a perfect accuracy of 87% [26,29]. Ekert et al. assessed panoramic images for the presence of periapical lesions with the help of CNN. They concluded that different tooth types are difficult to assess on panoramic image in different ways because of the radiographic image generation process. This is why the diagnostic may be uncertain and the sensitivity needs to be improved, although the results of periapical lesion detection by neural network have been satisfactory. In molars, the CNN’s sensitivity was higher (87%) than on other teeth, whereas the specificity was lower [26]. Artificial neural networks may not only be used in dental radiology, but also in genetics as it comes to endodontics [30]. In the study of Poswar et al., artificial intelligence was used to analyze the gene expression for radicular cysts (RCs) and periapical granulomas (PGs). The results showed that not only the inflammation, but also other biological processes may individuate the RCs and PGs because of their different gene expression [31].

Neural networks in endodontics may be useful in X-ray analysis and mostly in the detection of periapical lesions. This detection process can still be improved to obtain good accuracy for all teeth. Artificial intelligence might also be used in non-radiological areas such as genetics or others to ease the diagnosis. 

## 4. Neural Networks in Orthodontics

Artificial intelligence is spreading widely in the field of orthodontics. The most often used types of algorithms in orthodontics are artificial neural networks (ANN), convolutional neural networks (CNN), support vector machine, and regression algorithms [32]. Peilini et al. used an ANN in their study to predict whether patients need extractions or not in their treatment plan. Moreover, they took the anchorage patterns into consideration. The accuracy of the artificial neural network in the success of the treatment plan was 94.0% for extractions and 92.8% in the prediction of the use of maximum anchorage. These results indicate that ANN can be used by orthodontists to make more precise treatment plans [33]. Auconi et al. developed a system based on artificial neural networks with the purpose to predict the treatment outcomes in class II and III patients. The analysis could anticipate the co-occurrence of auxological anomalies during individual craniofacial growth and possibly localize reactive sites for a therapeutic approach to malocclusion [34,35]. The research indicates that the deep learning neural networks might be the best for TMJ osteoarthritis detection. Temporomandibular joint (TMJ) disorders are the second most common musculoskeletal condition affecting 5 to 12% of the population, and chronic disability in TMJ osteoarthritis (OA) increases with age. The main goal is to diagnose the impairment of the TMJ before morphological degeneration occurs. To achieve this goal in Bianchi et al.’s research, TMJ CBCT scans, serum, and saliva tests were taken [36,37,38]. In the study by Muraev et al., ANN was used to place the cephalometric points on cephalometric radiography. The accuracy of CP placement was compared between the ANN and three groups of doctors: expert, regular, and inexperienced. The results showed that ANN had a similar accuracy in planning cephalometric points as an experienced dentist and in some cases, they can be even more precise than new doctors [39]. In addition, ANN may help in the determination of the growth and development periods. In the research by Kök et al., the cephalometric and hand-wrist radiographs were obtained from patients aged between eight and 17 years. The growth-development periods and gender were determined from the cervical vertebrae by using ANN and the accuracy value of the results was found to be 94.27% [40]. 

To resume, the most common fields of orthodontics where neural networks may be used are in diagnosis and treatment planning, automated anatomic analyses, assessment of growth and development, and the evaluation of treatment outcomes (Table 3) [32]. It seems that artificial intelligence in orthodontics may be widely used and its use for sure can be extended even further. 

## 5. Neural Networks in Dental Surgery

According to the found literature, neural networks may be widely used in dental surgery. The purpose of Chien-Hsun Lu et al.’s study was to evaluate and improve post-orthognathic surgery image predictions for the individual patient. Simulations made by neural networks may be helpful for surgeons, orthodontists, and for the patients to improve the treatment plans [41]. The research of Patcas et al. indicated that artificial intelligence may characterize the impact of orthognathic surgery on facial attractiveness and age appearance. Pre- and post-treatment photographs of orthognathic patients were collected and convolutional neural networks were trained on >0.5 million images for age estimation and with >17 million ratings for attractiveness. According to the algorithms, most patients’ appearance improved with treatment (66.4%), resulting in a younger appearance of nearly one year. The same author used convolutional neural network to assess the attractiveness of patients who had undergone cleft surgeries [42,43]. In Byung Su Kim et al.’s work, convolutional neural networks were used to predict whether third molar extraction may lead to paresthesia of the inferior alveolar nerve. Extraction of the lower third molar is one of the most popular dental surgery procedure. The paresthesia of the nerve after mandible wisdom tooth extraction is quite a common complication. The panoramic images were used before the extraction and the anatomical relationship between the nerve canal and dental roots was used by the CNN to predict the occurrence of nerve paresthesia. However, the authors concluded that two dimensioned images as panoramic radiographs may lead to more false positive and false negative results, therefore, future research is needed [44]. Deep learning can be beneficial in odontogenic lesion detection. Two common diseases that might occur in jaws, and especially in the posterior ramus and body of the mandible, are ameloblastoma (AB) and odontogenic keratocyst (OK). In Liu et al.’s research, panoramic radiographs were used to detect these two tumors due to the lower cost and better accessibility than CT or MR images. Since it is difficult for human eyes to identify AB and OK in panoramic radiographs, a convolutional neuron network based on the transfer learning algorithm was used. The radiographs were especially prepared to obtain better contrast in the region of interest. All of the lesions were confirmed by the histopathological examination. The accuracy of the convolutional neural network was 90.36%, which was a better result than the accuracy of three other neural networks used in the same research. The above study indicates that neural networks may be useful to oral maxillofacial specialists before surgery [45].

According to previous studies, neural networks may be used in implantology. Dental implant treatment planning with the usage of three-dimensional cone-beam computed tomography (CBCT) images can be facilitate by AI systems [46]. Moreover, convolutional neural networks can be used to identify dental implant brands on panoramic radiographs and to identify the stage of treatment [47]. The quality of the osteointegration can be assessed by using convolutional neural networks (Table 4). The difficulties in osteointegration might occur due to the presence of a soft tissue layer (non-mineralized bone tissue) around the bone–implant interface, which can be exposed upon ultrasound examination [48]. Finally, artificial intelligence has been used in studies to measure the peri-implant bone loss [49].

Neural networks in dental surgery might be widely used in many areas starting with the orthognathic surgeries, changes in the bones or post extraction complications, and ending with implantology treatment. In particular, implantology is an area that is developing very rapidly and the use of neural networks might be very helpful in daily practice because of the need for high precision and meticulous planning. Neural networks may also help to predict some complications that may occur during surgical treatment and therefore avoid some of them.

## 6. Neural Networks in Periodontology

Periodontitis is a wide spread disease that concerns billions of people worldwide and if untreated, leads to tooth mobility and in severe cases, to tooth loss. To prevent this from happening, early disease detection and effective therapy needs to be carried out. To obtain reliable diagnosis, a meticulous physical examination needs to be performed. For this reason, dental probing to measure pocket depth and clinical attachment loss is performed. Periodontal probing has limited accuracy because of the individual examiner’s assessment. Commonly used additional examinations are dental radiographs, whose evaluation also depends on the examiner’s experience. To minimize errors in diagnosis, some authors have used neural networks. Krois et al. evaluated panoramic radiographs with the help of convolutional neural networks to detect periodontal bone loss in percentage of the tooth root length. The results were compared with the measures made by six experienced dentists. The CNN had higher accuracy (83%) and reliability than the dentists (80%) in detecting periodontal bone loss [50]. Peri-implant bone loss can be detected on dental periapical radiographs, but the difficulty is that the margins of bone around the implants are usually unclear or the margins can overlap. For this reason, convolutional neural networks can assess the marginal bone level, top, and apex of implants on dental periapical radiographs. In the study by Jun-Young Cha et al., the bone loss percentage was calculated and classified by the automated system. This method can be used to assess the severity of peri-implantitis [51]. In the research of Lee et al., a deep convolutional neural network was used to analyze the radiographs and measure the radiographic bone loss (RBL) for each tooth. RBL percentage, staging, and presumptive diagnosis according to the new periodontitis classification made by CNN were compared with the measurements made by independent examiners. The accuracy for the neural network was 85%. Thus, neural networks may be useful tools to assess radiographic bone loss and to obtain image-based periodontal diagnosis [49]. Other authors have also used neural networks to evaluate radiographic bone loss, and in this way, developed an automatic method for staging periodontitis according to the new criteria proposed at the 2017 World Workshop on the Classification of Periodontal and Peri-implant Diseases and Conditions. Chang et al. used panoramic images and convolutional neural networks to detect the periodontal bone level (PBL), the cementoenamel junction level (CEJL), and the teeth, and in this way, made a diagnosis of periodontitis stage [52]. Vadzyuk et al. took into consideration the psychological features to predict the development of periodontal disease. They concluded that patients’ level of anxiety and stress hormone levels had an impact on periodontitis (Table 5). Assessment of the condition of teeth hard tissues, the level of oral hygiene, and the evaluation of psychophysiological features with the use of neural networks can effectively predict the risk of periodontal disease development in young people [53]. 

The use of neural networks in periodontology can be a helpful tool for clinicians in daily practice as well as for scientists. The precise assessment of bone loss can be crucial in making periodontal diagnosis and treatment planning. More research and improvements are needed to introduce this tool into everyday periodontal use.

## 7. Conclusions

Dentistry is a field of medicine where new technologies are developing very quickly. Nowadays, artificial intelligence and neural networks are mostly used in dental radiology to facilitate diagnosis, treatment planning, and prediction of the treatment results. Other areas of dentistry where neural networks are used are genetics, psychology, microbiology, and many others. The most frequently used types of neural networks are artificial neural networks and convolutional neural networks. In restorative dentistry, neural networks can detect tooth decay or restorations, moreover, they can facilitate the choice of caries excavation method [3,22,23]. In endodontics, neural networks can be useful in detecting periapical lesions and root fractures, root canal system anatomy evaluation, predicting the viability of dental pulp stem cells, determining working length measurements, and predicting the success of retreatment procedures [25,26,27,28,29]. In orthodontics, they can facilitate the diagnosis and treatment planning, cephalometric points marking, anatomic analyses, assessment of growth and development, and the evaluation of treatment outcomes [33,34,36,39,40]. In dental surgery, neural networks may be helpful in orthognathic surgery planning, prediction of post-extraction complications, bone lesion detection, and differentiation and implantological treatment planning [41,42,43,44,45,46,47]. Furthermore, artificial intelligence is spreading into periodontology and in the above-mentioned studies, it was used to evaluate the periodontal bone loss, peri-implant bone loss, and to predict the development of periodontitis due to the psychological features [49,50,51,52,53,54]. This review shows that artificial intelligence has developed very fast in recent years and it may become an ordinary tool in modern dentistry in the near future. The advantages of this process are better efficiency, accuracy and precision, better monitoring, and time saving [55]. More research is needed with the use of neural networks in dentistry to put them into daily practice and to facilitate the work of dentist. 

## Figures and Tables

**Table 1:** Baseline characteristics of the studies included in the review by studying neural networks in restorative dentistry.

Study [Ref.]	Year of Publication	Type of Data	Type of Neural Network	Number of Database	Accuracy of Neural Network
Javed [3]	2019	Primary molars	Artificial neural network (ANN)	45 teeth	99.03%
Geetha [22]	2020	Periapical radiographs	Back -propagation neural network	105 images	97.7%
Abdalla-Aslan [23]	2020	Panoramic radiographs	Cubic support vector machine algorithm with error-correcting output codes	83 images	93.6%

**Table 2:** Baseline characteristics of the studies included in the review by studying neural networks in endodontics.

Study [Ref.]	Year of Publication	Type of Data	Type of Neural Network	Number of Database	Accuracy of Neural Network
Saghiri [25]	2012	Teeth	ANN	50 teeth	96%
Ekhert [26]	2019	Panoramic radiographs	CNN	-	87% (molars)
Setzer [27]	2020	CBCT images	Deep Learning	20 images	93%
Orhan [28]	2020	CBCT images	CNN	153 images	92.8%
Pauwels [29]	2021	Periapical radiographs	CNN	-	87%

**Table 3:** Baseline characteristics of the studies included in the review by studying neural networks in orthodontics.

Study [Ref.]	Year of Publication	Type of Data	Type of Neural Network	Number of Database	Accuracy of Neural Network
Auconi [33]	2015	Cephalometric records	Fuzzy clustering repartition	54 cephalograms	83.3%
Peilini [34]	2019	Medical records	ANN	302 patients	94.0% (extraction pattens); 92.8 % (anchorage patterns)
Bianchi [36]	2020	CBCT blood serum saliva clinical investigation	Logistic Regression, Random Forest, LightGBM, XGBoost	52 patients	82.3%
Muraev [39]	2020	Cephalometric records	ANN	330 cephalograms	99.9%
Kök [40]	2021	Cephalometric and hand-wrist radiographs	ANN	419 patients	94.27%

**Table 4:** Baseline characteristics of the studies included in review by studying neural networks in dental surgery.

Study [Ref.]	Year of Publication	Type of Data	Type of Neural Network	Number of Database	Accuracy of Neural Network
Chien-Hsun Lu [41]	2009	Profile photographs	ANN	30 patients	84.5%
Patcas [42]	2018	Photographs	CNN	146 patients	-
Patcas [43]	2019	Frontal and profile images	CNN	20 patients	-
Byung Su [44]	2021	Panoramic radiographs	CNN	300 images	82.7%
Liu [45]	2021	Panoramic radiographs	CNN	420 images	90.36%
Bayrakdar [46]	2021	CBCT	CNN (Diagnocat)	75 images	72.2% for canals detection; 66.4% for sinuses/fossae and 95.3% for missing tooth regions
Sukegawa [47]	2021	Panoramic radiographs	CNN	9767 images	81.83%

**Table 5:** Baseline characteristics of the studies included in review by studying neural networks in periodontology.

Study [Ref.]	Year of Publication	Type of Data	Type of Neural Network	Number of Database	Accuracy of Neural Network
Lee [49]	2018	Periapical radiographs	CNN	1044 images	81.0% for premolars 76.7% for molars
Krois [50]	2019	Panoramic radiographs	CNN	353 images	81%
Chang [52]	2020	Panoramic radiographs	CNN	340 images	93%
Vadzyuk [53]	2021	Survey (oral hygiene and nutrition) dental examination, psychological testing	ANN	156 students	-
Jun-Young Cha [51]	2021	Periapical radiographs	CNN	708 images	88.89%
